# Feasibility of a Food Delivery Intervention during Pregnancy in a Rural US Population: The PEAPOD Pilot Study

**DOI:** 10.3390/nu15040816

**Published:** 2023-02-05

**Authors:** Jean M. Kerver, Yash Khiraya, Janel M. Gryc, Joseph C. Gardiner, Sarah S. Comstock

**Affiliations:** 1 Department of Epidemiology and Biostatistics, Michigan State University, East Lansing, MI 48824, USA; 2 Department of Food Science and Human Nutrition, Michigan State University, East Lansing, MI 48824, USA

**Keywords:** pregnancy diet, fiber intake, nutrition, pragmatic diet intervention, pregnancy biospecimen collection, gut microbiome

## Abstract

Pregnancy nutrition is important for maternal and child health and may affect the development of the infant gut microbiome. Our objective was to assess the feasibility of implementing a food-based intervention designed to increase fiber intake among pregnant women in a rural setting. Participants were enrolled (*N* = 27) mid-pregnancy from a prenatal care clinic in rural Michigan, randomized to intervention (*N* = 13) or usual care (*N* = 14), and followed to 6 weeks postpartum. The intervention was designed to be easily replicable and scalable by partnering with hospital foodservices and included non-perishable high fiber foods and recipes, as well as weekly delivery of salads, soup, and fresh fruit. Surveys, maternal blood, urine, and stool were collected at 24- and 36-weeks gestation and at 6 weeks postpartum. Infant stool was collected at 6 weeks. Participants were 100% White (7% Hispanic White, 7% Native American and White); 55% with education < 4-year college degree. Data on dietary intake and urinary trace elements are presented as evidence of feasibility of outcome measurement. Retention was high at 93%; 85% reported high satisfaction. The intervention described here can be replicated and used in larger, longer studies designed to assess the effects of pregnancy diet on the establishment of the infant gut microbiome and related health outcomes.

## 1. Introduction

Diet interventions designed to increase fiber intake have been shown to alter the gut microbiome in adults [1], but have not been rigorously tested during pregnancy. Maternal gut microbiome composition during pregnancy and/or lactation may influence infant gut microbiome development during a critical window of time, potentially leading to long-term health effects [2,3,4,5,6]. To test the effects of maternal dietary change on the development of the infant gut microbiome, pragmatic strategies for implementing dietary interventions that can be applied in both rural and urban settings are needed.

The range of dietary intervention strategies that have been employed to promote healthy dietary intake during pregnancy has recently been reviewed [7], but these interventions are often developed to assess behavioral change, not for testing the effects of dietary composition on maternal or infant biomarkers and health outcomes. Even fewer have been conducted in rural settings where unique challenges exist [8]. 

Rural regions have been characterized by limited access to quality healthcare, education, and transportation, contributing to inequities in healthcare outcomes compared with their urban counterparts [9]. Access to health care in rural areas of the US has been declining, in part due to the large number of rural hospital closures [10]. In our own work, we have shown that access to maternal and prenatal care services in rural Michigan is limited [11]. The hospital affiliated with the prenatal care clinic where we recruited for this study has the only neonatal intensive care unit in a 10,000 square mile region and has a large and entirely rural catchment area. This region is designated by the census as a rural “unique underserved population” because the entire region meets the definition for both a health professional shortage area and a medically underserved area/population. 

The overall goal of the Pregnancy EAting and POstpartum Diapers (PEAPOD) pilot study was to gather information to effectively refine an intervention so that it can be tested in a larger, longer study using a factorial design to assess the separate and combined effects of maternal diet during pregnancy and lactation on the establishment of the infant gut microbiome. The specific objectives were to test methods of randomization and outcome measurement and to assess the feasibility and acceptability of implementing the intervention and the study procedures, which included collecting survey data and multiple biospecimens (blood, urine, and stool) at multiple locations (prenatal clinic, telephone, and home), via multiple modes (interviewer-administered and self-collection). We present results indicating that this type of pragmatic food-based diet intervention is a feasible approach to use during pregnancy.

## 2. Materials and Methods

### 2.1. Recruitment

Pregnant participants were recruited from a single prenatal care clinic serving a rural population in the northwest region of Michigan’s Lower Peninsula. Inclusion criteria were maternal age of at least 18 years, pregnant with a gestational age of approximately 22 weeks at enrollment, and no self-reported contraindication to increasing dietary fiber. Inclusion criteria were broad in an attempt to enroll a sample generally representative of the area, but we did restrict enrollment to those women living within a three-county region because of the food delivery component. All participants met in-person with a research assistant to complete the informed consent process and receive oral and written directions about the next steps of the study. The PEAPOD study was approved by the Institutional Review Boards at Michigan State University (#16-1515) and Munson Medical Center (#1026493). 

### 2.2. Intervention and Usual Care

**Figure 1:**
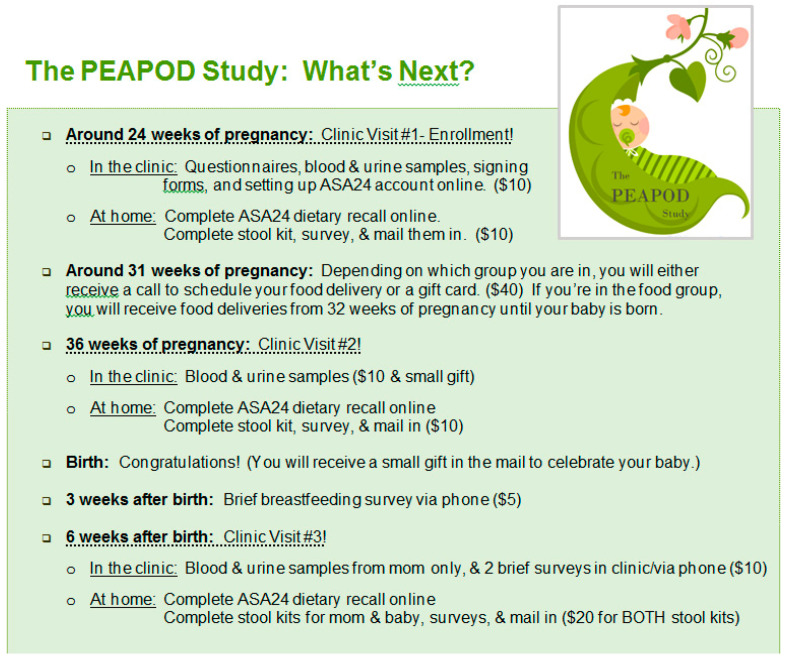
Study flyer with timeline and incentive structure.

A two-arm randomized controlled trial design was implemented with women enrolled in mid-pregnancy (*N* = 27). Participants were randomly assigned to the intervention (*N* = 13) or to usual care (*N* = 14) and followed to 6 weeks postpartum. The randomization process was led by the study biostatistician (J Gardiner) and included a block randomization schema [12] with study arm assignment provided in sealed, labeled envelopes that were opened and recorded in a sequential manner so that study personnel had no opportunity to influence study arm assignment. Study enrollment and baseline interviewer-administered data collection occurred at the prenatal care clinic at approximately 24 weeks gestation. Dietary data, described in more detail below, were ascertained via an automated self-administered system via computer, smart phone, or tablet at home after the enrollment visit. The intervention was initiated for those assigned to the intervention arm at 32 weeks gestation and continued until the infant’s birth, although “post-intervention” data collection occurred at 36 weeks gestation to increase the likelihood of complete data even if the participant delivered preterm. In the initial week of the intervention phase, participants in the intervention arm received non-perishable high fiber foods (whole wheat cereal, oatmeal, dried fruit, and canned beans) as well as olive oil, vinegar, recipes for salad dressing and side dishes, and general nutrition information. Partnering with a hospital catering service, the intervention included weekly food delivery of 3 large, prepared green salads, 2 quarts of soup including either legumes or whole grains (e.g., beans or barley), and 5 pieces of fresh fruit (e.g., apples or oranges). The food content of the intervention was primarily designed by one of the study principal investigators (J Kerver), a registered dietitian, in consultation with the partnering registered dietitians on the hospital foodservice team. Participants in the usual care arm were provided only a monetary benefit of $40. See Figure 1 for an overview of the participant timeline and incentive structure. 

### 2.3. Diet Assessment

Maternal diet was assessed before (i.e., 24-weeks gestation) and 4 weeks after the initiation of the intervention (i.e., 36-weeks gestation), and at 6 weeks postpartum. Participants were asked to complete self-administered 24-hour dietary recalls utilizing the 2016 version of the Automated Self-Administered 24-hr (ASA-24) Dietary Assessment Tool developed by the US National Cancer Institute [13]. The ASA-24 Tool is described in detail elsewhere [13], but briefly, it is designed to allow researchers to provide an electronic link to the web-based system with easy-to-follow instructions for participants to complete on their own. There were no specialized instructions provided to the participants by study personnel with dietary assessment expertise, which allowed us to test the feasibility of using this system for future studies with larger sample sizes and minimal research staff involvement. The study protocol called for participants to complete one weekday and one weekend 24-hr dietary recall at each data collection time point (i.e., 24- and 36-weeks gestation, and 6 weeks postpartum), and they were given these instructions verbally at their study enrollment visit and provided with written instructions to take home. 

### 2.4. Survey and Biospecimen Collection, Storage, and Analyses

Data collection included surveys (including sociodemographic and behavioral information as well as the ASA-24 described above), maternal blood, urine, and stool collection at three time points (24- and 36-weeks gestation [pre- and 4-weeks post-intervention initiation, respectively]; and at 6 weeks postpartum), and infant stool at age 6 weeks. Blood and urine specimens were obtained in the prenatal clinic and immediately aliquoted and stored at –80 °C. Fecal samples were self-collected at home and sent to the laboratory by mail. Fecal aliquots were stored at –80 °C upon reaching the lab. The average time between home sample collection and laboratory receipt was 3.8 ± 1.9 days (median = 3.5 days). 

We utilized the laboratory services provided by the National Institutes of Health Children’s Health Exposure Analysis Resource (CHEAR) Pilot and Feasibility (P&F) Program to measure plasma carotenoids and lipids, as well as urinary heavy metals and urinary metabolites. Trace elements reported in this manuscript were assessed from urine samples collected at 36-weeks gestation and were measured using an Agilent 8800 Triple Quadrupole ICP-MS (Santa Clara, CA, USA). These elements were analyzed in part to determine if metals serve as a marker for the presence of environmental contaminants, but they are reported here to show the feasibility of outcome assessment. Stool sample DNA extraction and amplification were performed in the laboratory of one of the study principal investigators (S Comstock). 16SrRNA gene sequencing was performed at the Michigan State University Research Technology Support Facility Genomics Core. All stool microbiome methods used this previously described protocol [14]. 

## 3. Results

### 3.1. Participants 

Our sample was considered entirely rural, and maternal characteristics reflected the local population (Table 1): 100% White (including 7% Hispanic White, 7% Native American and White); 89% reported at least some college, but only 45% with a bachelor’s degree or higher; 26% were enrolled in the public insurance program, Medicaid; and 33% reported having ever smoked. Mean maternal age was 29.6 y (range 20–40 y) and mean pre-pregnancy BMI was 26.9 kg/m^2^ (range 18.5–41.6). The three-county region from which we recruited has a population of approximately 135,000 (US Census). This rural region reports predominantly white race (over 96%) and with a higher than national average Native American population (as high as 3.7% in Leelanau County; Data USA). The age of the population skews older with a median age of 43 years in Grand Traverse and Kalkaska Counties and 55 years in Leelanau County (US Census), which has some implications for maternal health care availability because the region generally caters to an older population. 

### 3.2. Feasibility and Acceptability of Implementing Study Procedures

Data collection adherence was high with few missing data points. Table 2 shows that at the first study visit, prior to intervention initiation, we had 100% completion for survey, blood, urine, and stool collection. At 36-weeks gestation, which was 4 weeks after the initiation of the intervention, we had 93% completion for stool collection and 96% completion for blood and urine collection. At 6 weeks postpartum, we had 93% completion for survey, blood, urine, and stool. 

Overall satisfaction was high, with 85% reporting satisfied or very satisfied, and important qualitative insights were gained from participants. Participants who completed the final study visit (*N* = 25) reported contentment with partaking in the study and were willing to recommend the study to other individuals. Open-ended survey responses indicated that a small group of usual care participants (*N* = 6) used their gift card incentives to purchase healthier groceries. The participants reported reliable communication and suggested an increased variety of dietary options during intervention (Table 3). 

### 3.3. Dietary Intake Data 

Per protocol, participants were instructed to self-complete two 24-hour dietary recalls using the ASA-24 online system at three different time points (24- and 36-weeks gestation, and 6 weeks postpartum). Table 2 summarizes the number of dietary recalls that were completed per timepoint and Table 4 shows selected nutrient and food group data for those participants who had at least one dietary recall at each of the 24- and 36-weeks gestation data collection time points (n = 22). At baseline/24-weeks gestation, the number of participants with at least one dietary recall was 12 in the intervention arm and 13 in the usual care arm, with five participants in each group completing both dietary recalls as directed. At 36-weeks, the number of participants with at least one dietary recall was 11 each in the intervention and usual care arms, with 10/11 participants in the intervention arm and 9/11 participants in the usual care arm completing both dietary recalls as directed. Of note is that several participants completed additional dietary recalls as directed (i.e., one weekday and one weekend), but not until after the intervention began, so those data were excluded. At 6 weeks postpartum, complete dietary data were available for only seven participants in the intervention arm and six in the usual care arm. In Table 4 we present selected nutrient and food group data from the ASA-24 dietary data system for those participants who had at least one valid dietary recall at both the pre- and post-intervention data collection time points to show descriptive evidence of feasibility of outcome measurement. Following best practices for pilot studies with small sample sizes, we are not including statistical testing between study arms [15,16], although we did assess differences within each study arm pre- vs. post-intervention. The only difference that approached statistical significance was an increase in the dietary intake of fiber in the intervention arm between the pre- and post-intervention time periods. The mean (SD) was 18.0 (6.1) g fiber at 32 weeks gestation vs. 22.7 (7.8) g fiber at 36 weeks gestation, *p* = 0.052 using a two-tailed Student’s *t*-test for a paired comparison. 

### 3.4. Urinary Trace Elements

Descriptive measures of trace element concentrations assessed from urine samples collected at 36-weeks gestation are reported in Table 5. Generally, there was high variability in the levels of trace elements in the urine samples collected from pregnant women at 36-weeks gestation. Because our aim was to test the feasibility of outcome ascertainment and our sample size was too small to make inferences about the source population, we do not make comparisons to national data here. However, US nationally representative data are available for urinary measures of trace elements, and they have been reported elsewhere in comparison to similar measurements made among pregnant women in Puerto Rico and the US Pacific Northwest [17,18]. In those studies, predictors of trace element biomarkers included fish, public water, and dietary supplement consumption, which are relationships that could be examined in studies with larger sample sizes using the methods described here. 

### 3.5. Results Published To-Date

In this publication, we focus on the feasibility and acceptability of data, but there are both ongoing and previously published findings from the biospecimen analyses resulting from this pilot study. Data published elsewhere reported the positive association between dietary and plasma carotenoids with alpha diversity in the maternal fecal microbiota collected during the third trimester of pregnancy [19]. In addition, dietary consumption of pro-vitamin A carotenoids was associated with significantly different gut microbiome composition compared with that of participants who consumed lower amounts of such carotenoids. Eighty-five percent (n = 23) of participants had complete data, including plasma carotenoid measures, dietary intake data, and fecal microbiome data. These pregnant women had recently consumed carotenoid-containing foods including oranges/orange juice (17%); fruits (83%); carrots, sweet potatoes, mangos, apricots, and/or bell peppers (48%); egg (39%); tomato/tomato-based sauces (52%); or vegetables (65%). Plasma carotenoid concentrations averaged 39.0 µg/dL trans-lycopene, 17.7 µg/dL β-carotene (BC), 6.4 µg/dL α-carotene (AC), 11.4 µg/dL cryptoxanthin, and 29.8 µg/dL zeaxanthin and lutein. Notably, plasma concentrations were indicative of recent dietary intake with AC and BC concentrations higher in women who recently consumed foods high in carotenoids, and CR concentrations higher in women who consumed oranges/orange juice. However, an important limitation of this study is that the microbiome results may reflect an effect of high fiber or improved overall dietary quality, rather than a specific effect of carotenoids. Future research should explore a carotenoid-heavy intervention to assess the influence of carotenoids on the microbiota relative to other vegetables or fruits while carefully controlling for total fiber intake. 

We have also shown that fecal bacterial communities differ by lactation status in postpartum women and their infants [20]. Though the influence of human milk on the infant gut microbiota has been extensively studied, the gut microbiota of lactating women has yet to be rigorously explored. The bacterial composition of stool samples collected from post-partum participants who were exclusively feeding their infants human milk was significantly different compared with that of other post-partum participants. However, these gut microbiome observations may be confounded by maternal body mass index. Future, larger trials will be well-suited to resolve this outstanding question. We observed the presence of the *Bifidobacterium longum* subspecies *longum* in the maternal gut microbiota and the infant gut microbiota. For infants with *Bifidobacterium longum* subspecies *longum* present in their gut microbiotas, stool samples collected from their mothers at 6 weeks post-partum also contained that species. This is potentially important, as breastfed infants have lower risk for atopic disease, diarrhetic episodes, and childhood obesity [21,22], which may be related to specific bacterial taxa present early in life. This work can inform future trials to answer questions about the impact of lactation status on maternal gut microbiota and health outcomes for maternal/infant dyads.

Finally, while it is well-known that diet affects the gut microbiota and the subsequent production of metabolites, many specific interactions and associations have yet to be characterized. This is especially the case during pregnancy, a time of great metabolic and physiological upheaval. We recently published data from the trial described herein showing associations between urinary metabolites, diet, and the gut microbiota in the third trimester of pregnancy [23]. Therein, we describe 13 significant correlations between microbial taxa and dietary intake, as well as nine significant correlations between microbial taxa and urinary metabolites. We report that pregnant individuals whose gut microbiota was dominated by *Bacteroides* were more likely to have consumed fats, sodium, or protein-rich foods, and that these individuals also had significantly reduced microbial diversity in their gut bacterial communities.

## 4. Discussion

The aim of this study was to test procedures, including methods of randomization and outcome measurement, to assess the feasibility and acceptability of implementing a pragmatic, scalable, and replicable food-based intervention that can be used for answering important research questions, especially pertaining to infant gut microbiome development. The purpose of reporting the results is to encourage other researchers to develop similar pragmatic food-based dietary interventions that may not have the same rigor as providing participants with 100% of their food intake—prepared, for example, in a metabolic kitchen—but would allow for lower cost testing of important diet-related research questions. As such, we report feasibility results for collecting a variety of survey data and biospecimens to indicate that this type of food-based diet intervention is a feasible approach to use for research during pregnancy in a rural population where access to health care is often a barrier to research participation.

In our study population, this intervention was feasible and acceptable. Participation rates were high (93–100%) for all study components except the ASA-24 dietary recall. Self-administration of dietary recall via the ASA-24 was not well-liked by participants (n = 6 out of 25 participants who completed the final study visit noted this in a free text response on the satisfaction survey). However, for this study, we used the 2016 version of the ASA-24, which has now been updated three more times (2018, 2020, and 2022), in part to address some of the user-friendliness issues in previous versions (https://epi.grants.cancer.gov/asa24/ (accessed on 25 January 2023)). From a researcher perspective, we demonstrated the feasibility of dietary assessment using the ASA-24 before and after implementing a food-based intervention during pregnancy as evidenced here by the nutrient and food group outcome data ascertainment.

In terms of the composition of the food-based intervention, participants recommended providing choices in food selection to increase consumption (n = 6 out of 12 intervention participants who completed the final visit noted a version of this in a free text response). Usual care participants used their gift card incentives to buy healthy foods (n = 6 usual care group participants noted a version of this in a free text response) potentially leading to group contamination, which may be relevant for future research, depending on the study objectives. 

The main limitation of this work is the small sample size. We were able to test study procedures, but we are not able to make any inferences about the effect of the food-based diet intervention. However, by combining the usual care and intervention groups into a single study population, we have been able to ascertain important associations between diet, metabolites, and both adult and infant gut microbiota composition and diversity. In some small studies, nutrition education has been shown to have an impact on clinically important biomarkers. For example, in a study of 31 patients with chronic disease, an intensive nutrition education model with group and individual visits emphasizing whole, plant-based foods, significant improvements were shown in BMI, blood pressure, and cholesterol levels after 8 weeks [24]. However, in that study, as in many similar studies, participants were not randomized (all received the intervention). Further, participants were patients who were motivated by a recent clinical diagnosis of metabolic disease. This is a different situation than that encountered by generally healthy pregnant women.

The scope of dietary Interventions designed specifically for pregnant women has recently been reviewed [7,25]. The vast majority of dietary interventions in pregnancy include either nutrition education only, or supplemental food or vitamin/mineral supplements provided for low-income and potentially malnourished women. The purpose of all reviewed studies was to improve nutrient intake during pregnancy, which is an important goal. However, in this study, we are reporting the feasibility of implementing a pragmatic, food-based dietary intervention that can easily be implemented anywhere co-located with a hospital foodservice. Our study design can inform others conducting research in community settings. Future, larger trials using the methods described herein will answer questions about the effects of diet during pregnancy on maternal and child health outcomes.

## 5. Conclusions

This pilot trial produced valuable information. The intervention was tested with 27 pregnant women residing in rural Michigan. Retention was high at 93%, with 85% of participants reporting high satisfaction. Among the parameters compared across or within study arms, no significant differences were observed in dietary intake, plasma carotenoid levels, urinary metabolites, or gut microbiota composition. However, this test case can be used to effectively refine practical food-based interventions so they can be tested in larger, longer studies using, for example, a factorial design to test the effects of pregnancy diet and/or postpartum diet of breastfeeding moms on the establishment of the infant microbiome.

## Figures and Tables

**Table 1:** Maternal characteristics at study enrollment.

Characteristic	*N*	%
Race/Ethnicity		
White	27	100
White + Hispanic/Latina	2	7
White + Native American	2	7
Education Level		
High school diploma or equivalency	3	10
Some college or Associate’s Degree	12	45
Bachelor’s Degree or higher	12	45
Medicaid health insurance (yes)	7	26
Living with baby’s father (yes)	27	100
Ever smoked	9	33
Body Mass Index Category		
Normal	14	52
Overweight or Obese	13	48
Selected Dietary Variables	Mean	SD
Dietary fiber (g)	20.3	7.6
Dietary fat (% kcal)	37.4	6.5

**Table 2:** Visit participation rates.

Location/Mode of Data Collection and Study Visit Time	*N*	%
Clinic (interviewer-administered survey, blood, urine)		
Enrollment/24 weeks gestation	27	100
36 weeks gestation	26	96
6 weeks postpartum	25	93
Telephone (interviewer-administered survey)		
3 weeks postpartum	27	100
Home self-collection (stool)		
Enrollment (required before randomization)	27	100
36 weeks gestation	25	93
6 weeks postpartum	25	93
Home self-collection (ASA-24 dietary recall)		
24 weeks gestation (weekday)	24	89
24 weeks gestation (weekend)	15	56
36 weeks gestation (weekday)	20	74
36 weeks gestation (weekend)	17	63
6 weeks postpartum (weekday)	18	67
6 weeks postpartum (weekend)	13	48

**Table 3:** Patient satisfaction (free-text responses).

Question	*N*	Response
Did you like participating in this study? (*N* = 25 completed the study)	25	YES
Would you recommend it to others?	25	YES
What did you like the best?	N/A	“Easy to do, good communication on next steps”
What would you do differently?	N/A	“…hate peas, would not eat,” “…more choices,” “Hardest part was ASA-24. It was tedious!”
Did the gift cards help you with anything?	N/A	“…healthy eating,” “…weekly groceries”

**Table 4:** Dietary intake pre- and post-intervention by study arm (*N* = 22).

	Intervention Arm (*N* = 11)				Usual Care Arm (*N* = 11)			
	32 Weeks Gestation		36 Weeks Gestation		32 Weeks Gestation		36 Weeks Gestation	
Nutrient or Food Group	Mean	SD	Mean	SD	Mean	SD	Mean	SD
Energy (kcal)	2140	505	2164	385	2124	519	2205	506
Total Fat (g)	89.1	30.4	87.3	26.7	88.8	20.3	86.2	18.1
Saturated Fat (g)	31.4	12.0	29.5	10.1	29.6	8.1	32.1	7.9
Monounsaturated Fat (g)	29.3	9.6	29.4	8.3	31.4	8.9	30.2	7.0
Polyunsaturated Fat (g)	21.5	9.8	21.6	10.3	21.0	5.5	17.2	4.5
Total Carbohydrate (g)	263.5	72.4	264.6	55.5	253.0	82.7	280.6	88.5
Sugar (g)	128.3	56.9	119.9	31.1	109.4	43.8	130.9	58.4
Fiber (g)	18.0	6.1	22.7	7.8	20.1	7.6	17.4	7.2
Dark Green Vegetables (cup equiv.)	0.21	0.23	0.32	0.47	0.40	0.45	0.23	0.26
Red/Orange Vegetables (cup equiv.)	0.39	0.32	0.47	0.28	0.33	0.28	0.34	0.25
Legumes (cup equiv.)	0.15	0.19	0.11	0.14	0.06	0.13	0.03	0.07
Total Grains (oz equiv.)	5.81	2.66	6.80	1.90	7.50	3.56	7.56	2.21
Whole Grains (oz equiv.)	0.83	0.90	1.89	1.01	1.22	1.53	0.83	1.04
Refined grains (oz equiv.)	4.97	2.74	4.91	1.55	6.28	2.37	6.73	2.19
Nuts/Seeds (oz equiv.)	0.80	0.79	1.46	1.12	1.10	0.91	0.84	1.06
Vitamin C (mg)	87.0	69.7	86.2	38.2	94.2	55.7	62.8	35.3
Vitamin A, RAE (mcg)	763.6	307.7	809.6	324.2	704.2	302.2	700.2	259.1
Retinol (mcg)	509.0	204.1	574.6	249.3	493.8	235.8	522.5	259.2
Carotene, beta (mcg)	2738.6	1887.5	2560.3	2020.9	2268.7	2142.0	1880.1	1536.1
Carotene, alpha (mcg)	570.5	784.4	447.9	433.4	412.1	495.9	390.4	567.7
Vitamin K, phylloquinone (mcg)	171.2	97.3	164.8	122.8	145.0	91.1	97.3	60.4

**Table 5:** Trace elements (μg/L) from urine specimens collected at 36-weeks gestation (*N* = 26).

	Mean	SD	Minimum	Median	Maximum
Mg	52,395	46,226	5820	43,500	155,000
Al	21.1	4.41	13.3	20.7	31.40
V	0.060	0.039	0.020	0.047	0.207
Cr	0.477	0.125	0.319	0.436	0.858
Mn	0.294	0.093	0.181	0.277	0.561
Co	0.683	0.753	0.061	0.516	3.81
Ni	1.57	1.39	0.33	0.97	5.52
Cu	13.8	9.67	2.91	9.48	33.40
Zn	243	221	37.5	174	989
As	6.55	11.22	0.76	2.83	57.50
Se	40.8	22.9	14.8	31.5	105.00
Mo	44.2	36.2	8.66	31.95	143.0
Cd	0.130	0.105	0.007	0.107	0.426
Sn	4.27	18.0	0.148	0.487	94.1
Sb	0.138	0.121	0.037	0.104	0.606
Cs	4.21	3.84	0.70	2.62	17.4
Ba	2.84	3.33	0.30	1.63	16.9
Tl	0.154	0.098	0.033	0.133	0.405
Pb	0.480	0.190	0.292	0.410	0.985

## Data Availability

Data are available upon request from the corresponding author.
